# Sirtuin-2 Protects Neural Cells from Oxidative Stress and Is Elevated in Neurodegeneration

**DOI:** 10.1155/2017/2643587

**Published:** 2017-05-28

**Authors:** Preeti Singh, Peter S. Hanson, Christopher M. Morris

**Affiliations:** ^1^Medical Toxicology Centre and NIHR Health Protection Research Unit in Chemical and Radiation Threats and Hazards, Newcastle University, Wolfson Building, Claremont Place, Newcastle NE2 4AA, UK; ^2^NIHR Biomedical Research Unit in Lewy Body Disorders, Newcastle University, Edwardson Building, Institute of Neuroscience, Newcastle upon Tyne NE4 5PJ, UK; ^3^NIHR Biomedical Research Centre in Ageing and Chronic Disease, Newcastle University, Biomedical Research Building, Campus for Ageing and Vitality, Newcastle upon Tyne NE4 5PJ, UK

## Abstract

Sirtuins are highly conserved lysine deacetylases involved in ageing, energy production, and lifespan extension. The mammalian SIRT2 has been implicated in Parkinson's disease (PD) where studies suggest SIRT2 promotes neurodegeneration. We therefore evaluated the effects of SIRT2 manipulation in toxin treated SH-SY5Y cells and determined the expression and activity of SIRT2 in postmortem brain tissue from patients with PD. SH-SY5Y viability in response to oxidative stress induced by diquat or rotenone was measured following SIRT2 overexpression or inhibition of deacetylase activity, along with *α*-synuclein aggregation. SIRT2 in human tissues was evaluated using Western blotting, immunohistochemistry, and fluorometric activity assays. In SH-SY5Y cells, elevated SIRT2 protected cells from rotenone or diquat induced cell death and enzymatic inhibition of SIRT2 enhanced cell death. SIRT2 protection was mediated, in part, through elevated SOD2 expression. SIRT2 reduced the formation of *α*-synuclein aggregates but showed minimal colocalisation with *α*-synuclein. In postmortem PD brain tissue, SIRT2 activity was elevated compared to controls but also elevated in other neurodegenerative disorders. Results from both in vitro work and brain tissue suggest that SIRT2 is necessary for protection against oxidative stress and higher SIRT2 activity in PD brain may be a compensatory mechanism to combat neuronal stress.

## 1. Introduction

The mammalian Sirtuin, SIRT2, is a nicotinamide adenine diphosphate (NAD^+^) dependent cytoplasmic protein and an orthologue to yeast Hst2p [1]. SIRT2, though predominantly a cytoplasmic protein, shuttles between the cytoplasm and nucleus depending upon the cell cycle stage [2]. Human SIRT2 deacetylates a number of cytoplasmic and nuclear proteins and thus is a key modulator of many cellular processes including cell cycle, cell motility, autophagy, metabolic homeostasis, myelination, apoptosis, antioxidant defence mechanisms, and tumorigenesis. Although all SIRTs are expressed in the brain, SIRT2 is the most abundant [3] and is expressed in nearly all brain regions with particularly high levels in myelin- producing oligodendrocytes (OL) [4, 5]. In the mouse, isoform 2 of SIRT2, SIRT2.2 is highly expressed in the adult brain and age related accumulation of SIRT2.2 is observed in both the mouse and human cortex [3]. SIRT2 regulates myelin formation by deacetylating alpha-tubulin (Lysine 40) in OL [4] and deacetylating Par-3 (protease activated receptor) in Schwann's cells [6]. While being a modulator of OL differentiation, SIRT2 is also suggested to have a role in regulation of neurite growth and neuronal motility in hippocampal neurones [7]. These findings indicate that SIRT2 influences axonal plasticity and plays an important role in maintenance of neuronal networks in the brain and hence may be involved in age related neurodegenerative disorders such as Parkinson's disease (PD).

Parkinson's disease is the most common neurodegenerative movement disorder [8, 9] and typically involves progressive loss of dopaminergic (DA) neurones in the substantia nigra (SN) and accumulation of cytoplasmic inclusions, Lewy bodies (LB) and Lewy neurites composed of alpha-synuclein (*α*-synuclein) [10]. PD is clinically characterised by tremor, rigidity, bradykinesia, postural instability, and other accompanying symptoms [11]. Given the role of SIRTs in fundamental cell processes and protection from age related changes, the role of SIRT2 has begun to be studied in neurodegeneration. Inhibition of SIRT2 in cellular and drosophila models of PD reduces *α*-synuclein mediated toxicity [12]. Rotenone treatment of rats which causes SN cell death appears in part to rely on SIRT2 with treatment leading to an elevation of SIRT2 in the SN leading to worsening motor impairment while inhibition of SIRT2 diminished striatal DA depletion and improved behaviour abnormality [13]. Conversely, reduction of SIRT2 causes cell death in neural PC12 cells [14] and also in BV2 microglia [15, 16], with increased SIRT2 activity rescuing microtubule dynamics in SH-SY5Y cells [17]. Ablation of SIRT2 in the brain however causes minimal effects and therefore modulation of SIRT2 activity may be important in the context of cellular and in particular neuronal stress [18].

Given the possible role of SIRT2 in PD, the present study evaluated the role of SIRT2 in oxidative stress mediated cell death and characterised its role in PD. The effects of overexpression of SIRT2 and inhibition of deacetylase activity of SIRT2 were determined in oxidative stress in SH-SY5Y cells using diquat or rotenone, which induce cellular and mitochondrial stress, respectively [19, 20]. The effect of SIRT2 on *α*-synuclein aggregate formation in toxin treated SH-SY5Y cells was also evaluated. We also determined the expression, activity, and localisation of SIRT2 in postmortem human brain tissue obtained from the patients with PD, PD with dementia (PDD), dementia with Lewy bodies (DLB), and Alzheimer's disease (AD).

## 2. Materials and Method

### 2.1. SH-SY5Y Cells

SH-SY5Y neuroblastoma cells were obtained from the European Collection of Cell Cultures (ECACC, Salisbury, UK) and cultured as described previously [20]. Cells were grown at 37°C in a humidified atmosphere of 95% air/5% CO_2_.

### 2.2. SIRT2 Overexpression and Toxin Treatment in SH-SY5Y Cells

**Figure 1:**
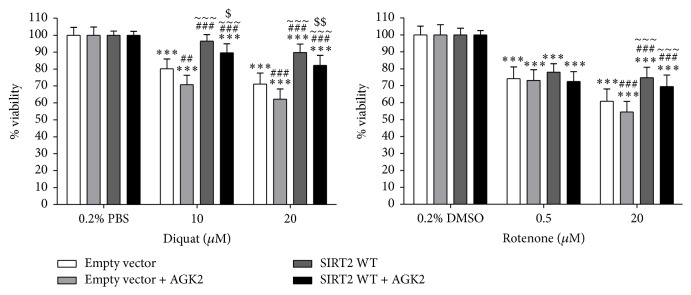
The effect of SIRT2 overexpression and inhibition was determined in toxin treated SH-SY5Y cells. SIRT2 was overexpressed in SH-SY5Y cells and control cells were transfected with empty vector following which one set of cells was treated with toxin alone and another with SIRT2 inhibitor AGK2 and toxin for 20 hours and viability measured by reduction of Alamar Blue. Toxins used: diquat (20 *µ*M or 10 *µ*M) or rotenone (20 *µ*M or 0.5 *µ*M) treated cells. Data are presented as fold- untreated (±SD) from three independent assays (*n* = 3). ^*∗∗∗*^*p* < 0.001 when compared to 0.2% vehicle (PBS or DMSO), one-way ANOVA (Bonferroni corrected), ^###^*p* < 0.001 and ^##^*p* < 0.01 when compared to control cells, ^~~~^*p* < 0.001 when compared to control + AGK2 treatment, and ^$$^*p* < 0.01 and ^$^*p* < 0.05 when compared to SIRT2 cells, two-way ANOVA (Bonferroni corrected).

**Figure 2:**
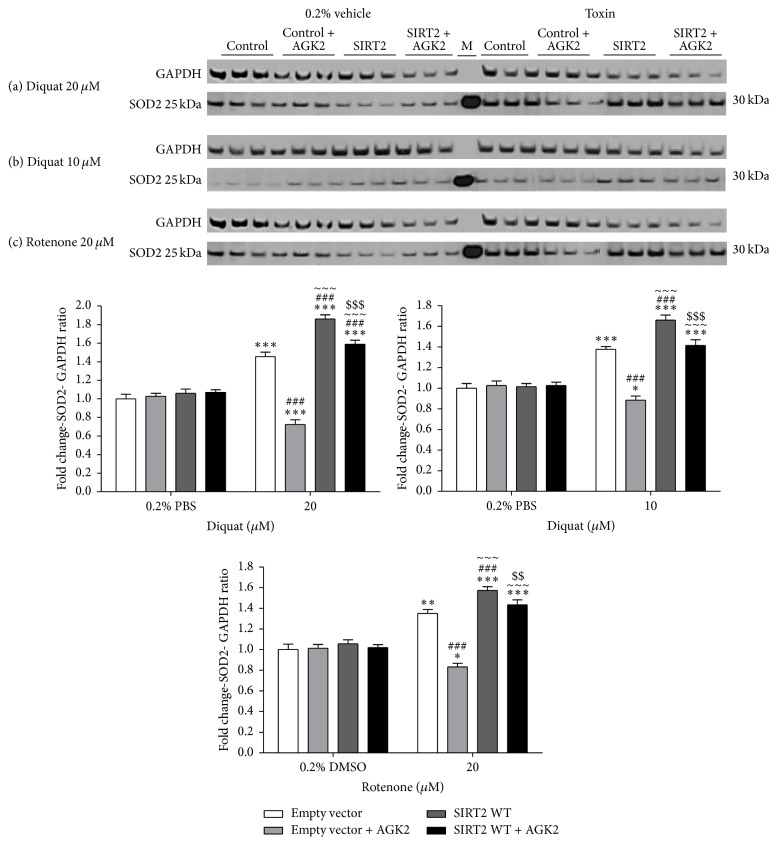
Expression of SOD2 was measured in toxin treated SH-SY5Y cells. SIRT2 was overexpressed in SH-SY5Y cells and control cells were transfected with empty vector following which one set of cells was treated with toxin alone and another with SIRT2 inhibitor AGK2 and toxin. Cells were harvested and the samples were probed for SOD2 expression. Data presented as fold-untreated (0.2% vehicle) (±SD) from three independent assays (*n* = 3). ^*∗∗∗*^*p* < 0.001, ^*∗∗*^*p* < 0.01, and ^*∗*^*p* < 0.05 when compared to 0.2% vehicle, one-way ANOVA (Bonferroni corrected), ^###^*p* < 0.001 when compared to control cells, ^~~~^*p* < 0.001 when compared to control + AGK2 treatment, and ^$$$^*p* < 0.001 and ^$$^*p* < 0.01 when compared to SIRT2, two-way ANOVA (Bonferroni corrected). M indicates molecular weight marker lane.

Wild type SIRT2 (SIRT2pcDNA3.1; Plasmid number 13813) was obtained from Addgene and pcDNA 3.1 was purchased from ThermoFisher Scientific. SH-SY5Y cells were seeded in 12-well plates and the cells were transfected with SIRT2pcDNA3.1 and the control group was transfected with empty pcDNA3.1 plasmid using PEI (polyethyleneimine; Invitrogen). Plasmids were incubated with cells at 37°C for 48 hours. To study the effect of SIRT2 inhibition, one set of cells transfected with SIRT2 and pcDNA3.1 were treated with either diquat (Sigma-Aldrich, UK) dissolved in PBS (Phosphate buffered saline; Sigma-Aldrich) or rotenone (Sigma-Aldrich) dissolved in DMSO (dimethyl sulphoxide, Sigma-Aldrich) at a final concentration of 0.2% PBS/DMSO alone and a second set of cells with toxin and AGK2 as a specific SIRT2 inhibitor (25 *μ*M; Tocris, UK; see Supplementary Figure 4 in Supplementary Material available online at https://doi.org/10.1155/2017/2643587). AGK2 was added to cells 2 hours prior to diquat or rotenone treatment and the cells were incubated overnight for 20 hours. Cell viability was determined by Alamar Blue reduction assay [20] (refer to Figures 1, 2, and 4 in supplementary files for efficiency of SIRT2 transfection and inhibition).

### 2.3. Western Blotting

Following toxicity determination, cell lysates were prepared by scraping the viable cells in native lysis buffer (1% 10x Tris buffered saline (TBS), 0.27 M Sucrose, 1% Triton X-100, 1x protease inhibitor cocktail). The cell lysates were sonicated for 20 seconds using a sonic probe and the total protein was determined using Bradford assay (modified from [21]). Twenty micrograms of protein in cell lysates were subjected to electrophoresis and were probed for selected antibodies as described previously [20] (see Table 1 in supplementary files for dilution and suppliers of antibodies).

### 2.4. Fluorescence Immunocytochemistry

SH-SY5Y cells were grown in chamber slides (BD Falcon, UK), transfected with SIRT2 plasmids, and treated with diquat or rotenone with or without AGK2. The cells were washed with PBS and slides incubated with 4% formaldehyde (Sigma-Aldrich) in warm 1x PBS for 15 minutes and then washed with PBS and stored until use in 10% glycerol (Sigma-Aldrich, UK) at 4°C. Cells were washed and blocked in 1x PBS/5% normal serum/0.3% Triton™ X-100 for an hour then incubated overnight at 4°C with SIRT2 and phospho-*α*-synuclein (Wako) for *α*-synuclein aggregates. Cells were washed with PBS and incubated with secondary antibodies for 60 minutes protected from light. Cells were washed with PBS and counterstained and mounted with ProLong Gold Antifade Mountant with DAPI (Thermo Fisher). Images were acquired using a Zeiss Axioplan 2 microscope (Zeiss, Oberkochen, Germany) with a 40x objective and images captured at 1024 × 1024 pixel resolution for analysis. Images were quantified using ImageJ (NIH, Bethesda, USA). The exposure time of the fluorescence was standardised to empty vector transfected cells with the same exposure time applied to all other sections with the SIRT2 overexpressing cells showing reduced *α*-synuclein staining intensity. All stained sections were quantified using ImageJ (NIH, Bethesda, USA) analysis of confocal images. The total immunostaining was analysed by importing the image to ImageJ and binarisation of the image (converted to 8-bit grey scale) and the highlighted cell area was quantified by using the “analyse particles” function. *α*-Synuclein aggregate immunoreactivity was also determined by using a standardised custom histogram based coloured thresholding technique and then subjected to “analyse particles.” The parameters recorded were total area and percentage area of staining. *α*-Synuclein aggregate percentage was calculated as the total area of *α*-synuclein divided by the total area of immunostaining multiplied by 100.

### 2.5. Postmortem Tissue Analysis

Brain samples were obtained from Newcastle Brain Tissue Resource, a Human Tissue Authority licensed tissue bank. All aspects of the study were approved by the National Research Ethics Service. Tissue was obtained at postmortem as soon as possible after death and samples were snap frozen and stored at −80°C. Frozen tissue of the relevant region was identified and protein homogenates from PD, DLB, PDD, AD, and controls (Table 1) were prepared by homogenising approximately 250 mg of freshly thawed grey matter in 2.5 ml of 0.2 M triethylammonium bicarbonate (TEAB) containing 1x protease inhibitor. After addition of 10 *µ*l of 10% SDS to 500 *µ*l of homogenate, samples were vortexed and then sonicated using a sonic probe for 15 secs, followed by sonication on ice in a sonic bath for 40 mins. The concentration of protein was determined by Bradford assay. Western blotting was performed as previously [20].

### 2.6. Sirtuin Activity

Brain protein homogenates were thawed and vortexed and sonicated as previous section. Samples were spun down at 100 g at 4°C for 5 minutes and the protein concentration of supernatant was determined by Bradford assay. Fluorescent SIRT substrate p53 (379–382), Ac-RHKK (Ac)-AMC was synthesised by Cambridge Research Biolabs, UK. Stock peptide was prepared as a 5 mM solution in diluted SIRT Assay buffer (50 mM Tris-HCl, pH 8.0, containing 137 mM sodium chloride, 2.7 mM KCl, and 1 mM MgCl_2_) and was stored at −70°C until use. Total SIRT activity was determined by using 30 *μ*g protein in substrate buffer containing 41.6 *µ*M peptide, 1 mM NAD^+^, and 100 nM TSA (as an HDAC inhibitor) and incubated at room temperature for 2 hours on a shaker. After 2 hours 2.5 *μ*g/ml trypsin in 50 mM NAM was added to stop further deacetylation and to cleave the deacetylated product. The fluorescence was recorded for each well after one hour of incubation of the trypsin-NAM solution in the plate reader on excitation wavelength of 350–360 nm and emission wavelength of 450–460 nm. SIRT2 activity was determined as AGK2 (20 *μ*M) inhibitable activity. Use of recombinant SIRT1, SIRT2, and SIRT3 showed equivalent activity with the Ac-RHKK (Ac)-AMC substrate (see supplementary files for sample and buffer preparation, and protein activity).

### 2.7. Determination of Cellular Localisation of SIRT2

Formalin fixed paraffin embedded brain tissue sections were used to determine distribution of SIRT2 in the CNS in disease. For immunohistochemistry, 10 *µ*M coronal sections were sampled from the temporal cortex, hippocampus, and cerebellum. The sections were heated at 60°C for 10 minutes followed by 2 × 10-minute washes in xylene (Fisher Scientific) followed by rehydration in decreasing ethanol solutions (2 × 100%, 95%, 70%, 50%, and 0% ethanol in ddH_2_O). Antigen retrieval was performed by boiling the sections in heated citrate buffer (pH 6) in a microwave at high power heat for 10 mins before allowing them to cool for 20 minutes and then washing them in running tap water. The sections were then quenched in 30% H_2_O_2_ in tap water for 20 minutes followed by 3 × 3-minute washes in TBS-T. Rabbit monoclonal antibody to SIRT2 (SantaCruz Biotechnology) dissolved in TBS-T was applied to the sections for an hour at room temperature followed by 3 × 3-minute washes in TBS-T. Sections were visualised using Menarini X-Cell Plus detection system according to the manufacturer's instructions with DAB reagent. Sections were counterstained with haematoxylin dehydrated through graded alcohols to xylene before coverslips were mounted with DPX (Fisher Scientific). Images of the sections were acquired using a Zeiss Axioplan 2 microscope (Zeiss, Oberkochen, Germany) with 10x and 63x magnifying objective and 3-chip CCD true colour camera (JVC, Yokohama, Japan) coupled to a PC.

### 2.8. Statistical Analyses

Statistical analysis was performed using one-way ANOVA within groups and two-way ANOVA within two groups using SPSS21 (IBM) followed by appropriate post hoc (Bonferroni) nonparametric testing. Error bars represent standard deviation (±SD). ^*∗*^*p* < 0.05 was considered statistically significant. Statistical analysis of Western blotting data was performed in GraphPad prism using a two-sample *t*-test assuming unequal variances. Statistical significance was considered as *p* < 0.05. The results are presented as mean ±SD.

## 3. Results

### 3.1. SH-SY5Y Cells

#### 3.1.1. Overexpression of SIRT2 Protects Cells from Toxin Mediated Cell Death

Diquat and rotenone have been reported to induce oxidative stress and rotenone has been shown to induce parkinsonian symptoms in a rat model [22]. Diquat is a potent redox cycler [23] that upon entry into cells utilises molecular oxygen to generate O_2_^−^ which can lead to lipid peroxidation in cell membranes resulting in cell death [24]. In diquat treated cells, overexpression of SIRT2 significantly increased viability compared to control cells (*p* < 0.001). A significant elevation in cytotoxicity was observed in control cells coincubated with diquat and AGK2 compared to diquat alone (20 *µ*M diquat: *p* < 0.001 and 10 *µ*M diquat *p* < 0.001). In cells treated with 0.5 *µ*M rotenone, no significant effect was seen by overexpression or inhibition of SIRT2 (*p* > 0.05) but in cells treated with 20 *µ*M rotenone, a reduction in toxicity was observed in SIRT2 overexpressing cells compared to control cells and to control cells treated with AGK2 (*p* < 0.001) (Figure 1).

#### 3.1.2. Under Oxidative Stress, SIRT2 Induces the Expression of SOD2

SIRT2 has been shown to increase antioxidant defence mechanisms by deacetylating FOXO3a and elevating FOXO3a DNA binding, resulting in an increased expression of SOD2 [25]. To test this possibility, the levels of SOD2 were measured in diquat or rotenone treated SH-SY5Y cells. In 20 *μ*M diquat treated cells, SOD2 levels were elevated in control (~1.5-fold, *p* < 0.001), SIRT2 (~2-fold, *p* < 0.001), and SIRT2 + AGK2 cells (~1.6-fold; *p* < 0.001) compared to 0.2% PBS treated control cells (Figure 2). The levels of SOD2 were reduced by 28% in control + AGK2 cells (*p* < 0.001) compared to 0.2% PBS treated control cells. In 10 *μ*M diquat treated cells, SOD2 levels were elevated in control (~1.5-fold, *p* < 0.001), SIRT2 (~1.7-fold, *p* < 0.001), and SIRT2 + AGK2 cells (~1.4-fold; *p* < 0.01) compared to 0.2% PBS treated control cells (Figure 2). The levels of SOD2 were reduced by 12% in control + AGK2 cells (*p* < 0.05) compared to 0.2% PBS treated control cells. The expression of SOD2 was tested only in 20 *μ*M rotenone treated cells, as 0.5 *μ*M rotenone cells did not show significant difference in cell viability between the groups (see Figure 1). In addition, at low levels of rotenone (0.5 *µ*M) oxidative damage may be limited to only the mitochondria leading to mitochondria-mediated cell death independent of cytoplasmic SIRT2. Higher levels of rotenone (20 *µ*m) with increased oxidative stress and mitochondrial inhibition leading to marked depletion of cellular ATP could reduce cytoplasmic SIRT2 phosphorylation causing increased SIRT2 activation [7] leading to induction of SOD2 and other mediators. The levels of SOD2 were elevated in control (~1.3-fold, *p* < 0.01), SIRT2 (~1.6-fold, *p* < 0.001), and SIRT2 + AGK2 cells (~1.4-fold; *p* < 0.001) compared to 0.2% DMSO treated control cells. The levels of SOD2 were reduced by 17% in control + AGK2 cells (*p* < 0.05) compared to 0.2% DMSO treated control cells (Figure 2).

#### 3.1.3. SIRT2 Shows Minimal Colocalisation with *α*-Synuclein

**Figure 3:**
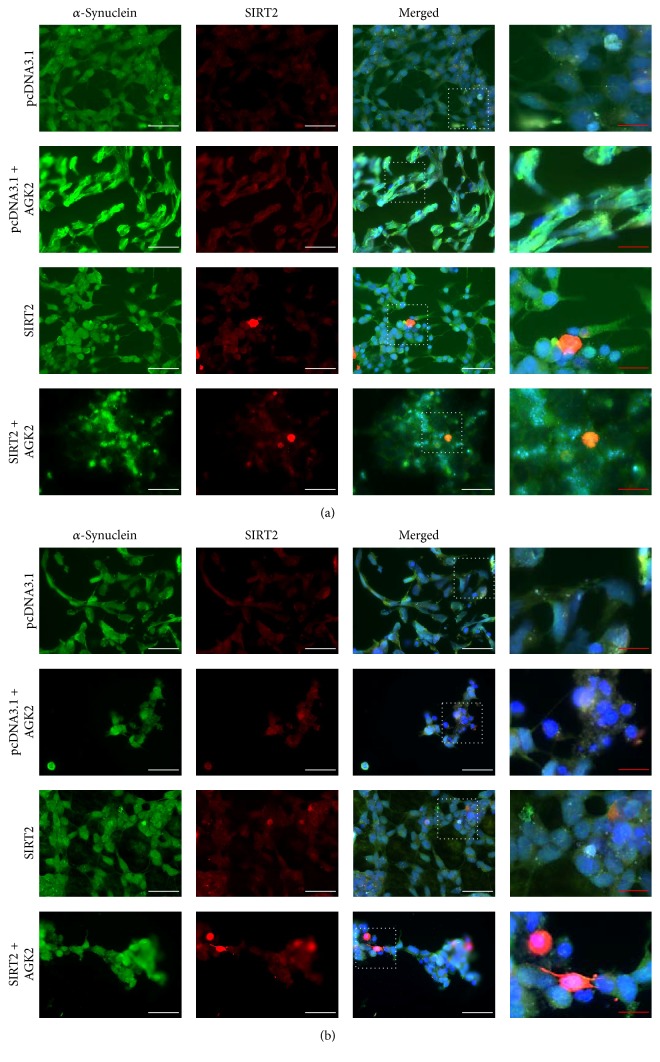
Localisation of SIRT2 and *α*-synuclein in diquat treated SH-SY5Y cells. Cellular distribution of SIRT2 and phospho-*α*-synuclein was determined using fluorescent immunocytochemistry. Images show *α*-synuclein immunostaining, SIRT2 immunostaining, and all staining merged including DAPI in 20 *μ*M diquat treated cells. Scale bars, white scale bar = 50 *μ*M and red scale bar = 20 *μ*M; magnification: 40x. (a) represents 0.2% PBS and (b) represents 20 *μ*M diquat treated SH-SY5Y cells.

**Figure 4:**
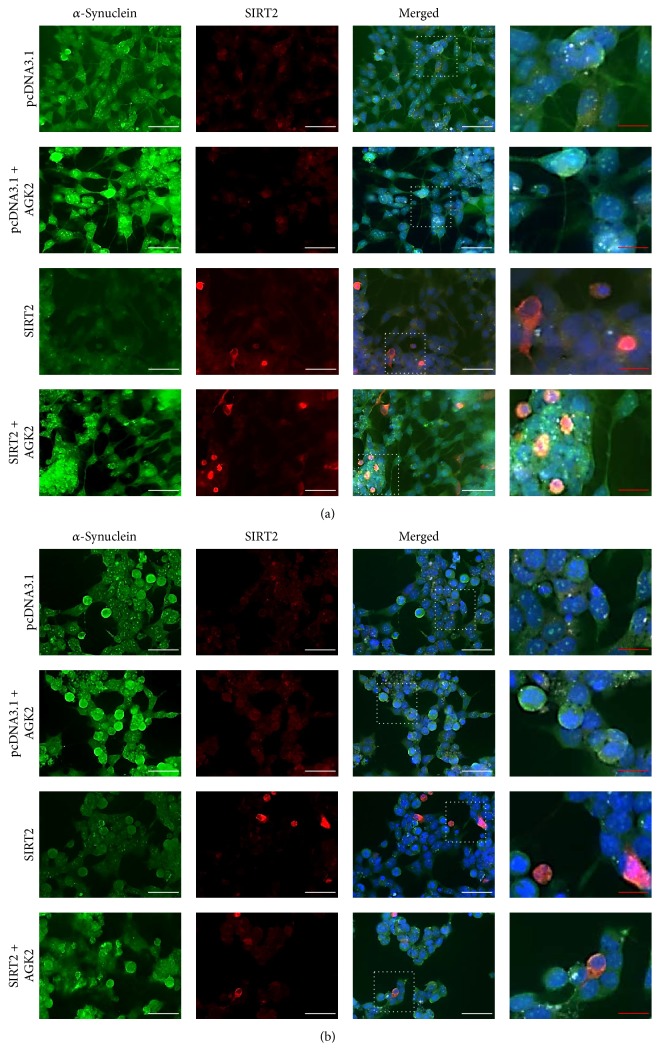
Localisation of SIRT2 and *α*-synuclein in rotenone treated SH-SY5Y cells. Cellular distribution of SIRT2 and phospho-*α*-synuclein was determined using fluorescent immunocytochemistry. Images show *α*-synuclein immunostaining, SIRT2 immunostaining, and all staining merged including DAPI in 20 *μ*M rotenone treated cells. Scale bars, white scale bar = 50 *μ*M and red scale bar = 20 *μ*M; magnification: 40x. (a) represents 0.2% DMSO and (b) represents 20 *μ*M rotenone treated SH-SY5Y cells.

Cells respond to stress by synthesising stress proteins and/or by relocalising the proteins to different cellular compartments. SIRT2 is a cytoplasmic protein which can translocate to the nucleus depending upon the cell cycle stage and cellular stress [2, 26]. To study the effect of cellular stress on localisation of SIRT2, SH-SY5Y cells were treated with diquat or rotenone to induce the stress and the localisation of SIRT2 was determined using immunocytochemistry and microscopy. On treatment with diquat or rotenone, SIRT2 was localised both in the nucleus and in the cytoplasm but was present prominently in the nucleus (see supplementary Figure 3). The localisation of SIRT2 in the nucleus under toxin induced oxidative stress could be attributed to the role played by SIRT2 in DNA damage repair and cell cycle regulation under normal circumstances and as well as under genotoxic stress [27, 28] (Figures 3 and 4).

#### 3.1.4. Inhibition of SIRT2 Enhanced *α*-Synuclein Aggregate Formation

**Figure 5:**
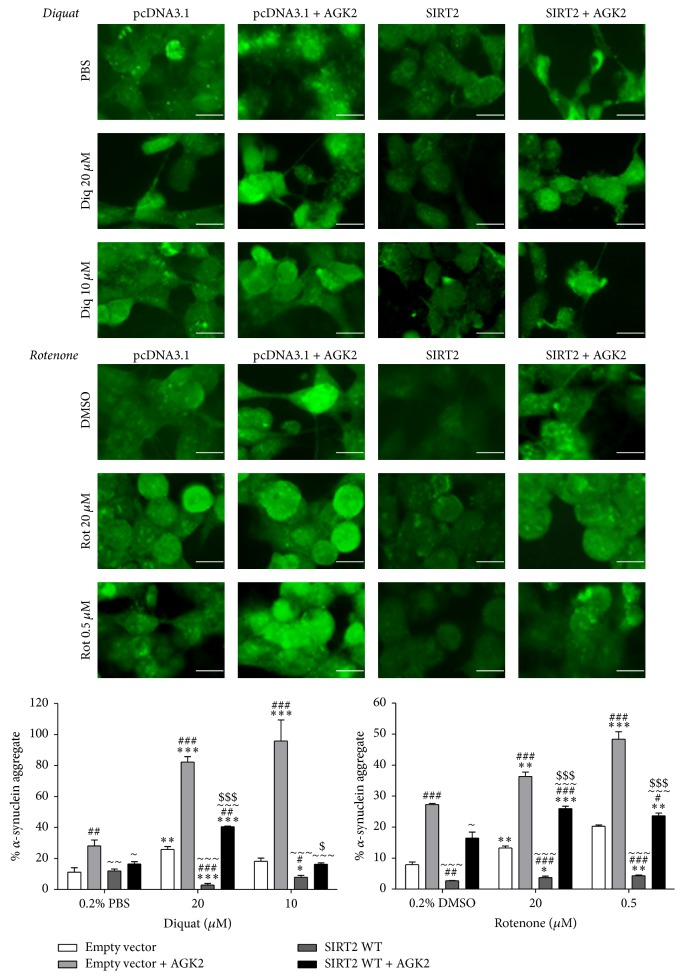
*α*-Synuclein aggregate formation and quantification in toxin treated SH-SY5Y cells. SIRT2 overexpressing SH-SY5Y cells were treated with toxin (20 *μ*M or 10 *μ*M diquat or 20 *μ*M or 0.5 *μ*M rotenone) and 0.2% PBS or DMSO; cells transfected with empty vector were used as a control and another set of SIRT2 and control cells was coincubated with AGK2 and toxin. Cells were immunostained with phospho-*α*-synuclein. Images were captured through GFP filter under 63x magnification. The captured images represent *α*-synuclein staining and the bar graphs represent the aggregate quantification in diquat or rotenone treated cells. Each bar represents % *α*-synuclein aggregates (±SD) from three independent assays (*n* = 3). ^*∗∗∗*^*p* < 0.001, ^*∗∗*^*p* < 0.01, and ^*∗*^*p* < 0.05 when compared to 0.2% vehicle, one-way ANOVA (Bonferroni corrected), ^###^*p* < 0.001, ^##^*p* < 0.01, and ^#^*p* < 0.05 when compared to control cells, ^~~~^*p* < 0.001, ^~~^*p* < 0.01, and ^~^*p* < 0.05 when compared to control + AGK2 treatment, and  ^$$$^*p* < 0.001 and ^$^*p* < 0.05 when compared to SIRT2 cells, two-way ANOVA (Bonferroni corrected). Scale bar: 20 *μ*M.

PD involves the progressive loss of DA neurones in the SN and the presence of LB rich in *α*-synuclein [10, 29]. The *α*-synuclein aggregates in LB are generally formed because of the association of misfolded *α*-synuclein proteins and the levels of misfolded proteins can increase under several conditions such as oxidative stress [30], inhibition of protein degradation [31], or mitochondrial dysfunction [32]. SIRT2 showed minimal colocalisation with *α*-synuclein suggesting that SIRT2 may not physically interact with *α*-synuclein (Figures 3 and 4). The minimal proportion of colocalisation may potentially be attributed to the interaction of both SIRT2 and *α*-synuclein with *α*-tubulin [33, 34]. The effect of SIRT2 on *α*-synuclein aggregate formation was determined in toxin and AGK2 treated SH-SY5Y cells. In diquat treated cells, a significant increase in aggregate formation was seen in AGK2 treated control cells when compared to 0.2% PBS treated control cells (*p* < 0.001). In SIRT2 + AGK2 cells, aggregate formation was higher than control (*p* < 0.01) and SIRT2 cells (*p* < 0.001) but was significantly lower than control + AGK2 (*p* < 0.001) cells when treated with 20 *μ*M diquat. Overexpression of SIRT2 inhibited *α*-synuclein aggregate formation in 20 *μ*M diquat treated cells compared to control cells (*p* < 0.001; <23%). In rotenone treated cells, AGK2 treated control (*p* < 0.001) and SIRT2 (*p* < 0.001) cells showed increased aggregate formation in all treatments with SIRT2 overexpression causing reduced aggregate formation (*p* < 0.001) (Figure 5).

#### 3.1.5. SIRT2 Protein in Neurodegeneration

**Figure 6:**
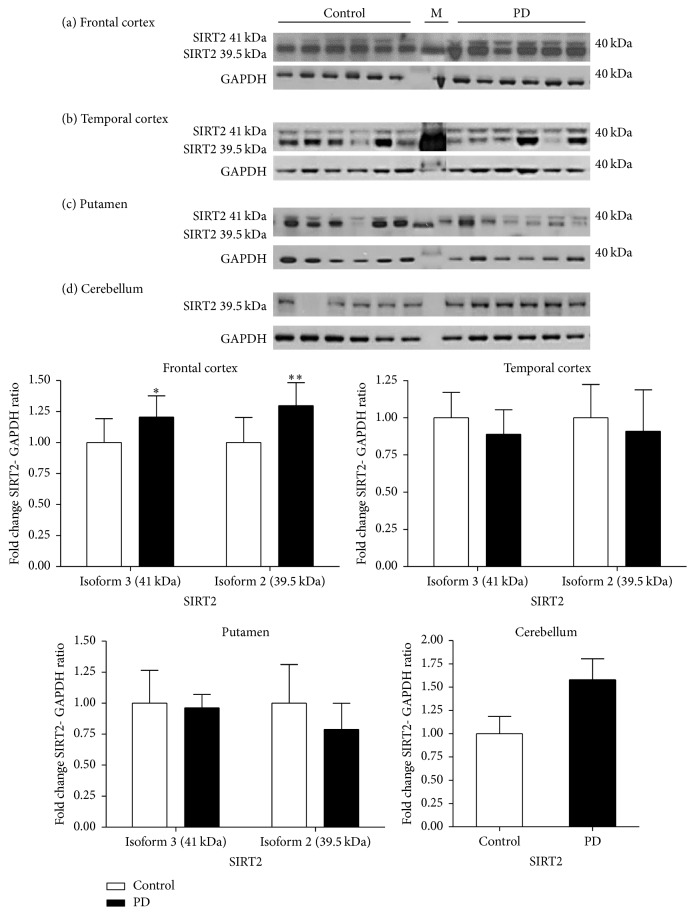
Expression of SIRT2 in different brain regions in Parkinson's disease. The levels of SIRT2 were determined in different regions of PD patients and were compared to a control-cohort. SIRT2 band intensity was normalised with GAPDH. Data are presented as fold change (±SD) with respect to control from three independent replicates. ^*∗∗*^*p* < 0.01, and ^*∗*^*p* < 0.05 when compared to control; statistical analysis was done through *t*-test performed on GraphPad prism. Images are representative blots of SIRT2 and GAPDH. M indicates molecular weight markers lane.

In the frontal cortex of PD cases, SIRT2.3 and SIRT2.2 isoforms were detected but not isoforms 1 and 4. Levels of SIRT2 were elevated, SIRT2.3 by 25% (*p* < 0.05) and SIRT2.2 by 30% (*p* < 0.01). In the temporal cortex, no significant difference was observed in the expression of either isoforms of SIRT2 between control and PD cases (*p* > 0.05). In the putamen, there was no observable difference in the level of SIRT2.3 whereas a reduction of 23% in the expression of SIRT2.2 was observed in PD but the difference was not statistically significant (*p* > 0.05). Western blot analysis of SIRT2 detected only SIRT2.2 in the cerebellum and an increase of 57% was noticed in PD compared to controls (*p* < 0.001) (Figure 6).

**Figure 7:**
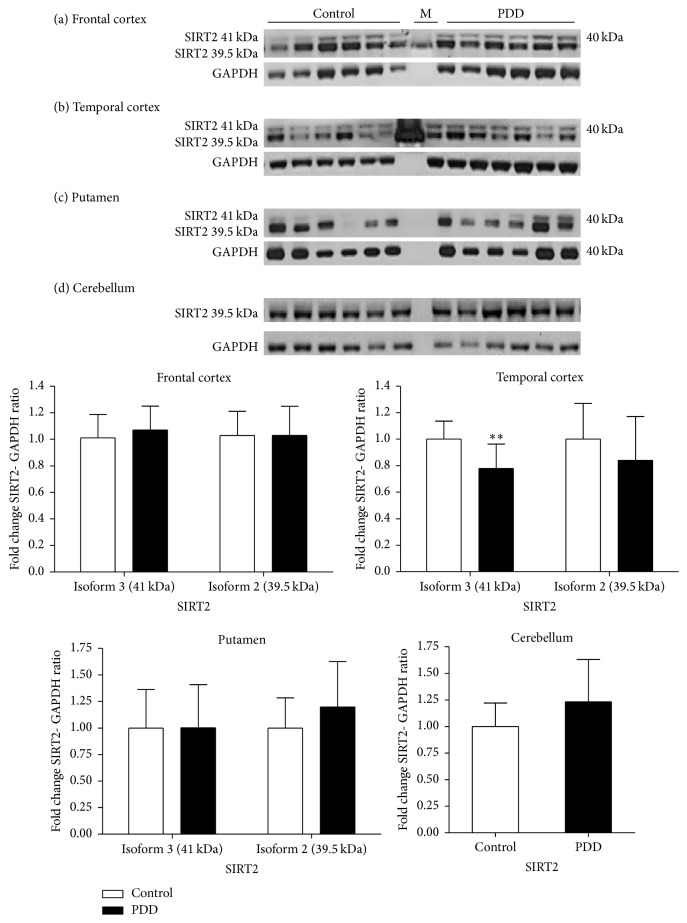
Expression of SIRT2 in different regions in Parkinson's disease with Dementia. The levels of SIRT2 were determined in different regions of PDD patients and were compared to a control-cohort. SIRT2 band intensity was normalised with GAPDH. Data are presented as fold change (±SD) with respect to control from three independent replicates. ^*∗∗*^*p* < 0.01 when compared to control; statistical analysis was done through *t*-test performed on GraphPad prism. Images are representative blots of SIRT2 and GAPDH. M indicates molecular weight marker lane.

In PDD, the levels of SIRT2 isoforms in the frontal cortex were not changed when compared to controls. In the temporal cortex, the levels of SIRT2.3 were reduced by 23% (*p* < 0.01) and a nonsignificant reduction of 16% was seen in SIRT2.2 levels (*p* > 0.05). In the putamen, no significant change in the levels of isoforms of SIRT2 in PDD compared to controls was observed (*p* > 0.05). Similar to PD, only SIRT2.2 was detected in cerebellar samples of PDD and no significant difference in the levels of SIRT2 was observed between PDD and control (*p* > 0.05) (Figure 7).

**Figure 8:**
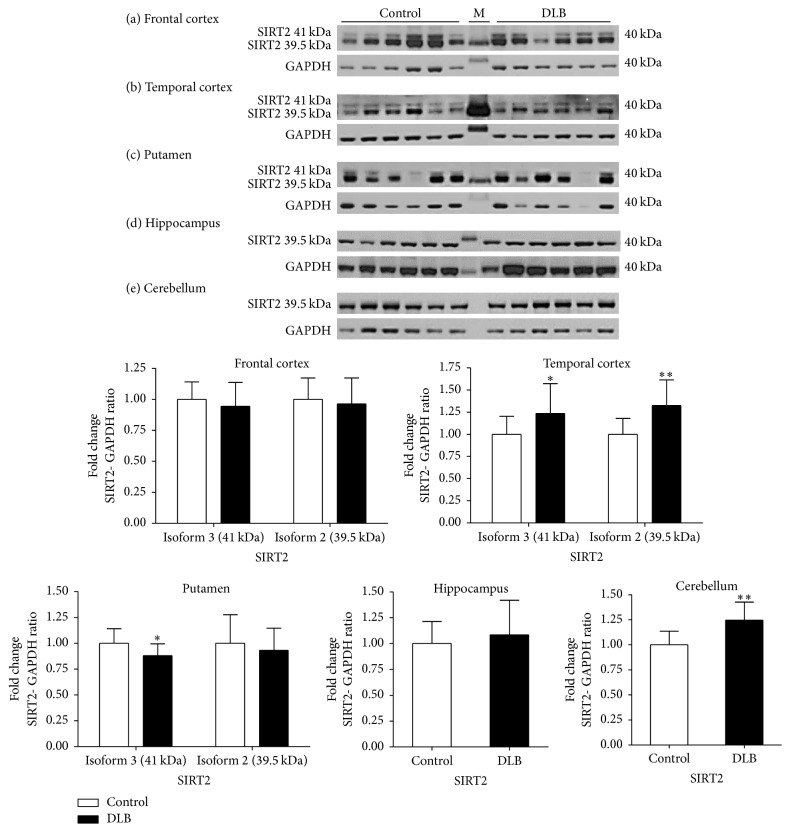
Expression of SIRT2 in different brain regions in dementia with Lewy Bodies. The levels of SIRT2 were determined in different regions of DLB patients and were compared to a control-cohort. SIRT2 band intensity was normalised with GAPDH. Data are presented as fold change (±SD) with respect to control from three independent replicates. ^*∗∗*^*p* < 0.01, and ^*∗*^*p* < 0.05 when compared to control; statistical analysis was done through *t*-test performed on GraphPad prism. Images are representative blots of SIRT2 and GAPDH. M indicates molecular weight marker lane.

In DLB frontal cortex samples, no significant difference was found in the levels of SIRT2 isoforms when compared to controls. In the temporal cortex of DLB patients, the levels of both the isoforms of SIRT2 were elevated, SIRT2.3 by 24% (*p* < 0.05) and SIRT2.2 by 33% (*p* < 0.01). The level of SIRT2.3 was significantly reduced in the putamen of DLB by 13% (*p* < 0.05) but no significant difference was observed in the level SIRT2.2. The levels of SIRT2.2 in hippocampal samples of DLB were not significantly changed and in cerebellar samples of DLB, the levels of SIRT2.2 showed an increase of 25% (*p* < 0.01) compared to control (Figure 8).

**Figure 9:**
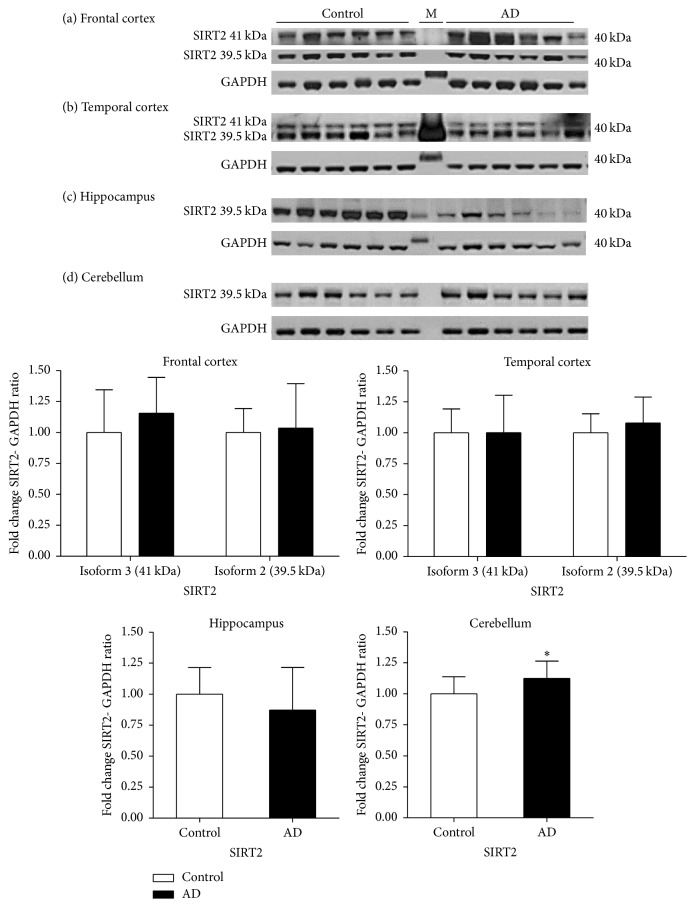
Expression of SIRT2 in different brain regions in Alzheimer's disease. The levels of SIRT2 were determined in different regions of AD patients and were compared to a control-cohort. SIRT2 band intensity was normalised with GAPDH. Data are presented as fold change (±SD) with respect to control from three independent replicates. ^*∗*^*p* < 0.05 when compared to control; statistical analysis was done through *t*-test performed on GraphPad prism. Images are representative blots of SIRT2 and GAPDH. M indicates molecular weight marker's lane.

In AD frontal cortex samples, no significant difference was observed in the levels of either isoforms of SIRT2 compared to controls. Similarly, no significant differences were observed in the levels of SIRT2 isoforms in the temporal cortex. The levels of SIRT2.2 in hippocampal samples were marginally reduced by 14% compared to controls but not significantly (*p* > 0.05). An increase of 14% was seen in the levels of SIRT2.2 in the cerebellum of AD compared to controls (*p* < 0.05) (Figure 9).

#### 3.1.6. SIRT2 Activity in Neurodegeneration

**Figure 10:**
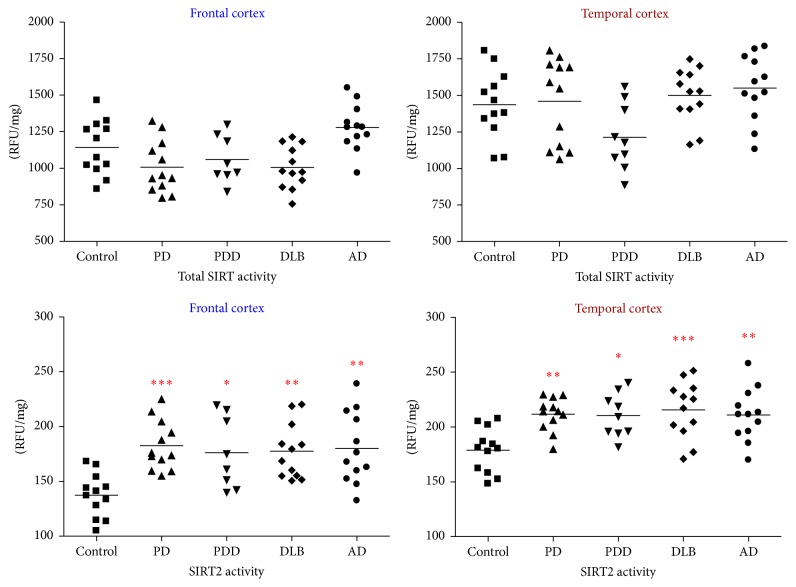
Total SIRT and SIRT2 activities in the frontal and temporal cortex in PD, PDD, DLB, AD, and controls. Total SIRT and SIRT2 activities were measured through a fluorometric enzymatic activity assay in the frontal and temporal cortices of PD, PDD, DLB, and AD patients and were compared to cohort-control group. ^*∗∗∗*^*p* < 0.001, ^*∗∗*^*p* < 0.01, and ^*∗*^*p* < 0.05 when compared to control, one-way ANOVA (Bonferroni corrected).

In the frontal cortex, measurement of total SIRT activity did not show any significant change between the disease groups and controls (*p* > 0.05); however, compared to AD, the total SIRT activity was reduced in PD and DLB by about 20% (*p* < 0.01) (though PDD did not show any significant difference). SIRT2 activity was upregulated in PD (33%; *p* < 0.001), PDD (28%; *p* < 0.05), DLB (29%; *p* < 0.01), and AD (31%; *p* < 0.01) compared to controls (*F* = 5.906, *p* < 0.001). In the temporal cortex, there was no significant difference in total SIRT activity between the disease groups and control (*p* > 0.05); however, compared to AD there was a significant reduction of 33% in total SIRT activity in PDD (*p* < 0.05), though other groups did not show any significant change. SIRT2 activity was upregulated in PD (19%; *p* < 0.01), PDD (17%; *p* < 0.05), DLB (21%; *p* < 0.001), and AD (18%; *p* < 0.01) compared to controls whereas no significant difference was seen among the disease groups (*F* = 5.593; *p* < 0.001) (Figure 10).

#### 3.1.7. Cellular Location of SIRT2 in Human Brain

**Figure 11:**
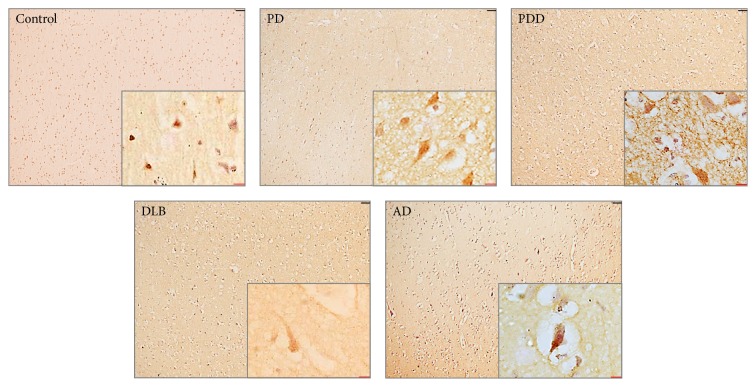
Cellular distribution of SIRT2 in the temporal cortex of disease and control groups. The images show the cellular localisation of SIRT2 in grey matter of superior temporal gyrus of temporal cortex in PD, PDD, DLB, AD, and control cases. SIRT2 was localised in both in the cytoplasm and the nucleus of the neurones in the groups, being predominantly present in the cytoplasm in AD. The picture in inset is 63x oil immersion image overlaid on 10x image. Scale bars, black scale bar = 50 *μ*M and red scale bar = 20 *μ*M.

The cellular localisation of SIRT2 was determined in the temporal cortex, hippocampus, and cerebellum in PD, PDD, DLB, AD, and an age matched control group. SIRT2 staining was weak and localised predominantly in the cytoplasm and nucleus of large pyramidal neurones in these groups whereas, in AD, SIRT2 was predominantly localised in pyramidal neurone cytoplasm. Staining was also apparent in the neuropil in all cases tested. In the temporal cortex, no apparent difference was observed within PD, PDD, DLB, and controls (Figure 11; Supplementary Figure 6). SIRT2 did not show a staining pattern that appeared to show cortical LB. These results suggest that there was no significant effect of disease conditions on the location of SIRT2 within the cell. The location of SIRT2 in different brain regions is summarised in Table 3 in supplementary files and the location of SIRT2 in other brain regions is shown in Figure 6 of supplementary files.

## 4. Discussion

Oxidative stress is a common general mechanism involved in cell death associated with neurodegenerative disorders [35] and has been implicated in the initiation and progression of PD [36]. Studies have reported that inhibition of SIRT2 reduces *α*-synuclein mediated toxicity in PD models and SIRT2 inhibition also rescued cells from mutant HTT in HD models [12, 37]. In contrast, the findings from this study suggest that reduced SIRT2 activity increased cell death following oxidative stress and increasing SIRT2 protects SH-SY5Y cells and is in agreement with other studies on SIRT2 [14, 17]. As with other SIRTs, SIRT2 is NAD^+^ dependent and is a potential redox sensor and studies have shown that SIRT2 enhances cell viability under oxidative stress and inhibition or knock-down of SIRT2 decreases intracellular ATP levels and enhances cell death. SIRT2 can regulate oxidative stress by deacetylating FOXO3a and increasing expression of FOXO3a targets, namely, p27^Kip1^ and SOD2, and under severe oxidative stress SIRT2 enhances the expression of the proapoptotic protein Bim [25]. Consequently, through p27^Kip1^, SIRT2 promotes cell cycle arrest and reduces the amount of ROS via SOD2 [25]. In this study, the enhanced expression of SOD2 potentially via FOXO3a following SIRT2 overexpression may be one route by which SIRT2 confers cellular protection [25]. In PC12 cells, silencing or inhibition of SIRT2 by AGK2 led to decreased ATP levels and enhanced cell death via necrosis [14]. In contrast, Nie et al. showed that inhibition of SIRT2 rescued differentiated PC12 cells from H_2_O_2_ induced toxicity and silencing of SIRT2 reduced the levels of ROS following H_2_O_2_ treatment [38]. These findings corroborate with results from this study and suggest that the associated upregulation of SOD2 and SIRT2 can regulate cell viability and combat oxidative stress.

Cellular stress subjects proteins to a variety of modifications which affect the stability, activity, and even the localisation of proteins [39]. In this study it was observed that under relatively high levels of oxidative stress (20 *µ*M rotenone, 10 *µ*M, or 20 *µ*M diquat), SIRT2 was localised to the nucleus and cytoplasm but was prominently present in the nucleus. The absence of any effect of SIRT2 at low levels of oxidative stress caused by 0.5 *µ*M rotenone may be insufficient to cause major depletion of ATP and lead to activation of SIRT2 by dephosphorylation causing SIRT2 to translocate to the nucleus [7]. SIRT2 is normally a cytoplasmic protein but may shuttle to the nucleus based upon cell cycle stage and cellular stress [2, 26]. Under oxidative stress, the known target of SIRT2, FOXO3a, shuttles to the nucleus [40] and induces the expression of its target SOD2 and may directly activate SOD2 to counteract the effect of ROS [41]. The results from this study are consistent with previous studies that report that SIRT2 localises to the cell nucleus under stress. Our finding that there is no induction of SOD2 in cells overexpressing SIRT2 under basal conditions would indicate that, following transfer of SIRT2 to the nucleus during oxidative stress, SIRT2 may interact with nuclear transcription factors such as FOXO3a which may lead to the induced expression of protective proteins such as SOD2.

Given the role of *α*-synuclein aggregation in PD, we found that SIRT2 inhibition enhanced aggregate formation in diquat or rotenone treated cells with overexpression of SIRT2 reducing *α*-synuclein aggregate formation. This contrasts with previous studies which reported that SIRT2 inhibition rescues *α*-synuclein mediated toxicity [12]. This may be attributed to different treatment regimens with A53T mutant *α*-synuclein overexpression directly aggregating in cellular and fly models whereas, in this study, *α*-synuclein aggregate formation was induced by oxidative stress. The results obtained from SH-SY5Y cells overexpressing SIRT2 under basal conditions, or from cells where SIRT2 is inhibited under basal conditions, suggest that SIRT2 does not promote de novo *α*-synuclein aggregate formation; rather SIRT2 impairs the formation of new or larger *α*-synuclein aggregates. Viability assays also corroborate the finding that SIRT2 inhibition enhances cellular death and SIRT2 overexpression promotes cell survival. All these findings suggest that SIRT2 acts as a prosurvival factor under oxidative stress.

The ageing brain shows gene expression changes specifically in the levels of stress response genes, mitochondrial genes, and genes involved in synaptic function [42]. SIRT2 plays a crucial role in antioxidant defence mechanisms [25] and in DNA damage repair [43] and elevated levels of oxidative stress and DNA damage are observed in neurodegenerative disorders such as PD and AD. In this study, the levels of SIRT2 protein did not show major or consistent changes in the disease groups compared to controls, although a tendency towards elevation in the levels of SIRT2 was observed overall. Maxwell et al. showed that SIRT2 accumulates in the ageing brain [3] and this may possibly explain why major changes were not observed in SIRT2 levels in disease groups, with age related changes masking disease related changes. While protein levels of SIRT2 were essentially unchanged, enzymatic activity for SIRT2 showed SIRT2 activity was higher in disease groups than control but no significant difference was observed between neurodegenerative disorders. The current results in induced oxidative stress in SH-SY5Y cells show SIRT2 overexpression promotes cell survival and inhibits *α*-synuclein aggregate formation. It is possible that elevated SIRT2 activity in neurodegenerative disorders is a compensatory mechanism to counteract the effects of oxidative stress occurring due to ageing and disease processes. SIRT1 has been shown to be neuroprotective in different neurodegenerative disorders and we found that SIRT1 activity was remarkably reduced in disease groups (unpublished data) although the total SIRT activity did not differ among the groups. SIRT1 and SIRT2 have been observed to share some targets including p53, NF-кB, histones H3, and H4 and following down-regulation of SIRT1 activity it is safe to assume that SIRT2 activity is elevated to target specific proteins such as p53 and modulate apoptosis, neuroinflammation, and genomic stability. Also, in relation to the in vitro work, it is possible that SIRT2 is upregulated in order to induce the activity of antioxidant proteins including SOD2 and catalase. Combining the in vitro and postmortem human tissue studies it is possible that SIRT2 is required to combat oxidative stress and higher SIRT2 activity in human brain tissue is a compensatory mechanism. The cellular localisation of SIRT2 showed no significant difference within the disease groups and controls. Generally, the sections showed SIRT2 localisation to the cytoplasm but also nuclei of neurones, which may relate to the translocation of SIRT2 to the nucleus under stress, corroborating the cellular work. Future work with additional SIRT2 antibodies would be needed to determine the location of SIRT2 along with dual-immunostaining for known neurodegenerative disease biomarkers. The role of SIRT2 should be further investigated under chronic oxidative stress in additional neuronal models of PD.

## 5. Conclusion

In this study we have shown that, in SH-SY5Y cells, under diquat or rotenone induced oxidative stress, SIRT2 overexpression protected cells from oxidative damage and reduced *α*-synuclein aggregate formation. Also, the inhibition of SIRT2 by AGK2 under oxidative stress, elevated cell death and promoted *α*-synuclein aggregate formation. These findings suggest that SIRT2 promotes cell survival and may provide this protection through elevation of SOD2. In postmortem human brain tissue, SIRT2 protein expression did not differ between different disease groups (PD, PDD, DLB, and AD) and controls but the enzymatic activity of SIRT2 was higher in disease groups compared to controls. Based upon the findings from the in vitro work and postmortem brain tissue studies, elevated SIRT2 in neurodegenerative disorders could be a compensatory mechanism to combat oxidative stress.

## Supplementary Material

Supplementary Figure 1: Transfection efficiency of SIRT2 in toxin treated SH-SY5Y cells.
Supplementary Figure 2: AGK2 treatment and its effect on expression of α-tubulin in toxin treated SH-SY5Y cells.
Supplementary Figure 3: SIRT2 localisation following oxidative stress.
Table 1: Details of antibodies used in Western blotting of SIRTs transfected and toxin treated SH-SY5Y cells.
Table 2: Details of antibodies used in fluorescence immunocytochemistry of SIRTs transfected SH-SY5Y cells.
Supplementary Figure 4: Activity of AGK2 against Recombinant Sirtuins.
Supplementary Figure 5: SIRT activity assay with recombinant SIRTs using Fluorescent SIRT substrate p53 (379-382), Ac-RHKK(Ac)-AMC.
Supplementary Figure 6: Cellular distribution of SIRT2 in the Hippocampus and Cerebellum of Disease and Control Groups.
Table 3: Summary table presenting localisation of SIRT2 in different brain regions of PD, PDD, DLB, AD and control groups.

## Figures and Tables

**Table 1:** Details of brain samples used for Western blot and immunohistochemistry.

Groups	FCX	TCX	Cb	Pu	Hp	Age at death (years)	Tissue pH	PMD (hours)	Gender	
									M	F
Control (*N*)	11	12	12	12	8	77.5 ± 6.98	6.17 ± 0.34	19.9 ± 6.42	7	5
PD (*N*)	12	12	12	12	—	77.44 ± 7.03	5.85 ± 0.06	23.44 ± 9.72	8	4
PDD (*N*)	8	9	12	8	—	75.93 ± 5.38	6.19 ± 0.32	24.69 ± 11.38	9	3
DLB (*N*)	12	12	12	12	6	77.00 ± 5.35	6.32 ± 0.29	18.0 ± 8.58	9	3
AD (*N*)	12	12	12	—	9	80.37 ± 5.25	6.19 ± 0.33	19.84 ± 9.30	5	7

The table summarises the case details of brain samples used in Western blot analysis and immunohistochemistry. FCX: frontal cortex; TCX: temporal cortex; Cb: cerebellum; Pu: putamen; Hp: hippocampus; PMD: postmortem delay.

## Abbreviations

AD: Alzheimer's disease
DA: Dopamine
DLB: Dementia with Lewy bodies
DMSO: Dimethyl sulphoxide
FOXO: Forkhead box subgroup O
HD: Huntington disease
HDAC: Histone deacetylase
LB: Lewy bodies
NAD: Nicotinamide-adenine-dinucleotide
NAM: Nicotinamide
NF-*κ*B: Nuclear factor kappa B
O_2_^−^: Superoxide anions
OL: Oligodendrocyte
PBS: Phosphate buffered saline
PD: Parkinson's disease
PDD: PD with dementia
PEI: Polyethylenimine
ROS: Reactive oxygen species
SIRT: Sirtuin
SN: Substantia nigra
SOD: Superoxide dismutase
TBS/T: Tris buffered saline/Tween
TSA: Trichostatin A.
