# Supplementary material for: Sirtuin-2 Protects Neural Cells from Oxidative Stress and Is Elevated in Neurodegeneration
Source: Parkinsons Dis. 2017 May 28;2017:2643587. doi: 10.1155/2017/2643587 (PMC5467326; doi:10.1155/2017/2643587)
Supplement: Supplementary file 1 — Supplementary Figure 1: Transfection efficiency of SIRT2 in toxin treated SH-SY5Y cells. Supplementary Figure 2: AGK2 treatment and its effect on expression of α-tubulin in toxin treated SH-SY5Y cells. Supplementary Figure 3: SIRT2 localisation following oxidative stress. Table 1: Details of antibodies used in Western blotting of SIRTs transfected and toxin treated SH-SY5Y cells. Table 2: Details of antibodies used in fluorescence immunocytochemistry of SIRTs transfected SH-SY5Y cells. Supplementary Figure 4: Activity of AGK2 against Recombinant Sirtuins. Supplementary Figure 5: SIRT activity assay with recombinant SIRTs using Fluorescent SIRT substrate p53 (379-382), Ac-RHKK(Ac)-AMC. Supplementary Figure 6: Cellular distribution of SIRT2 in the Hippocampus and Cerebellum of Disease and Control Groups. Table 3: Summary table presenting localisation of SIRT2 in different brain regions of PD, PDD, DLB, AD and control groups. [file 2643587.f1.pdf]

Supplementary materials and figures

1. Efficiency of SIRT2 transfection

Western blot analysis of diquat treated SH-SY5Y cells showed a significant increase in SIRT2 levels in SIRT2 transfected cells with a 3-3.5 fold increase in vehicle (0.2% PBS) treated cells compared to the empty vector transfected cells ($p<0.001$ compared to both empty vector and empty vector+AGK2, Supplementary Figure **1**). Similar increase was seen in 10µM diquat treated cells, interestingly a small increase of 12% in SIRT2 levels was observed in SIRT2 transfected cells compared to SIRT2 transfected cells treated with AGK2 ($p<0.01$). Similar to diquat treated cells, the levels of SIRT2 increased by 2.5-2.75 fold in 20µM rotenone treated cells and by 4-4.5 fold in 0.5µM rotenone treated cells ($p<0.001$) compared to empty vector transfected cells (Supplementary Figure **1**).

Note: The endogenous SIRT2 level remained undetected in SH-SY5Y cells possibly due to low level of SIRT2 in SH-SY5Y cells and over-expression of SIRT2 only resulted in expression of Isoform 3 of SIRT2 (41kDa).

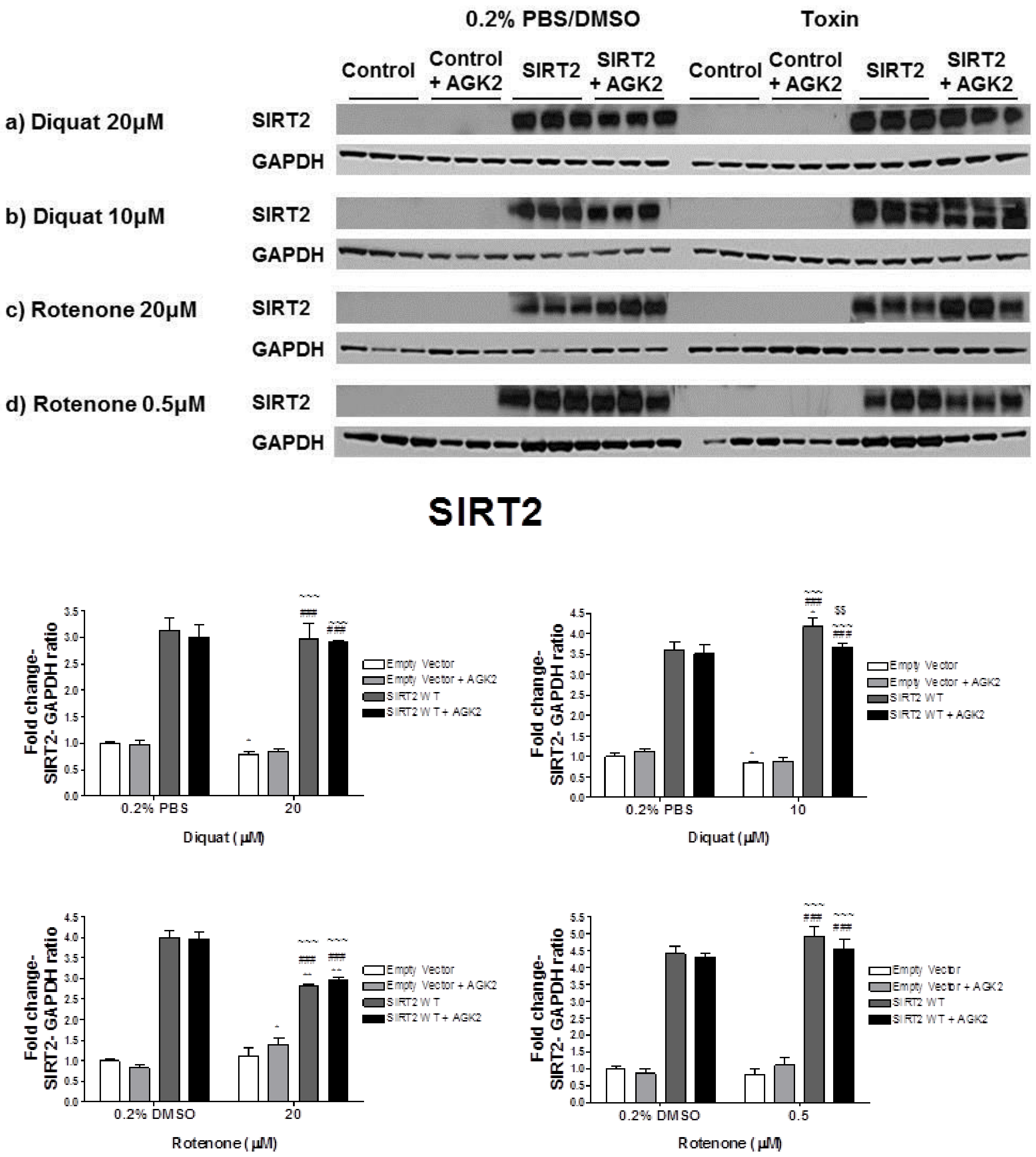

Supplementary Figure 1 Transfection efficiency of SIRT2 in toxin treated SH-SY5Y cells.

SIRT2 was over-expressed in SH-SY5Y cells and control cells were transfected with empty vector following which one set of cells was treated with toxin alone and another with SIRT2 inhibitor AGK2 and toxin. Cells were harvested and the samples were probed for SIRT2 expression; a-d show the representative blot of SIRT2 and GAPDH in cells treated with a) 20μM diquat, b) 10μM diquat, c) 20μM rotenone and d) 0.5μM rotenone. e-h represent the data are presented as fold- untreated (+SD) in cells treated with a) 20μM diquat, b) 10μM diquat, c) 20μM rotenone and d) 0.5μM rotenone. Data presented are from three independent assays (n=3). *p<0.05 when compared to 0.2% PBS/DMSO, one-way ANOVA (Bonferroni corrected), ###p<0.001and ##p<0.01 when compared to empty vector treatment, ~~~p<0.001 and ~p<0.05 when compared to empty vector + AGK2 treatment and $$$p<0.001 and $$p<0.01 when compared to SIRT2 overexpressing cells, two-way ANOVA (Bonferroni-corrected).

2. Efficiency of SIRT2 inhibition

In order to test the efficiency of SIRT2 inhibition by AGK2, levels of acetylated α-tubulin (K40), was measured in the samples. As shown in Supplementary Figure **2**, the levels of acetylated α-tubulin were elevated by 1.2-1.6 fold in cells co-incubated with AGK2 and diquat ($p<0.001$ in empty vector + AGK2 cells and $p<0.01$ in SIRT2+AGK2 cells) compared to empty vector transfected cells. The levels of acetylated α-tubulin were reduced by 50% in SIRT2 transfected cells ($p<0.01$; Supplementary Figure **2**) compared to empty vector transfected cells. These findings suggest that AGK2 is a potent SIRT2 inhibitor. The levels of acetylated α-tubulin were significantly higher in AGK2 treated cells in both rotenone treatments (20μM or 0.5μM rotenone: $p<0.001$) and the levels were reduced in SIRT2 transfected cells (20μM or 0.5μM rotenone: $p<0.001$; Supplementary Figure **2**).

Diquat

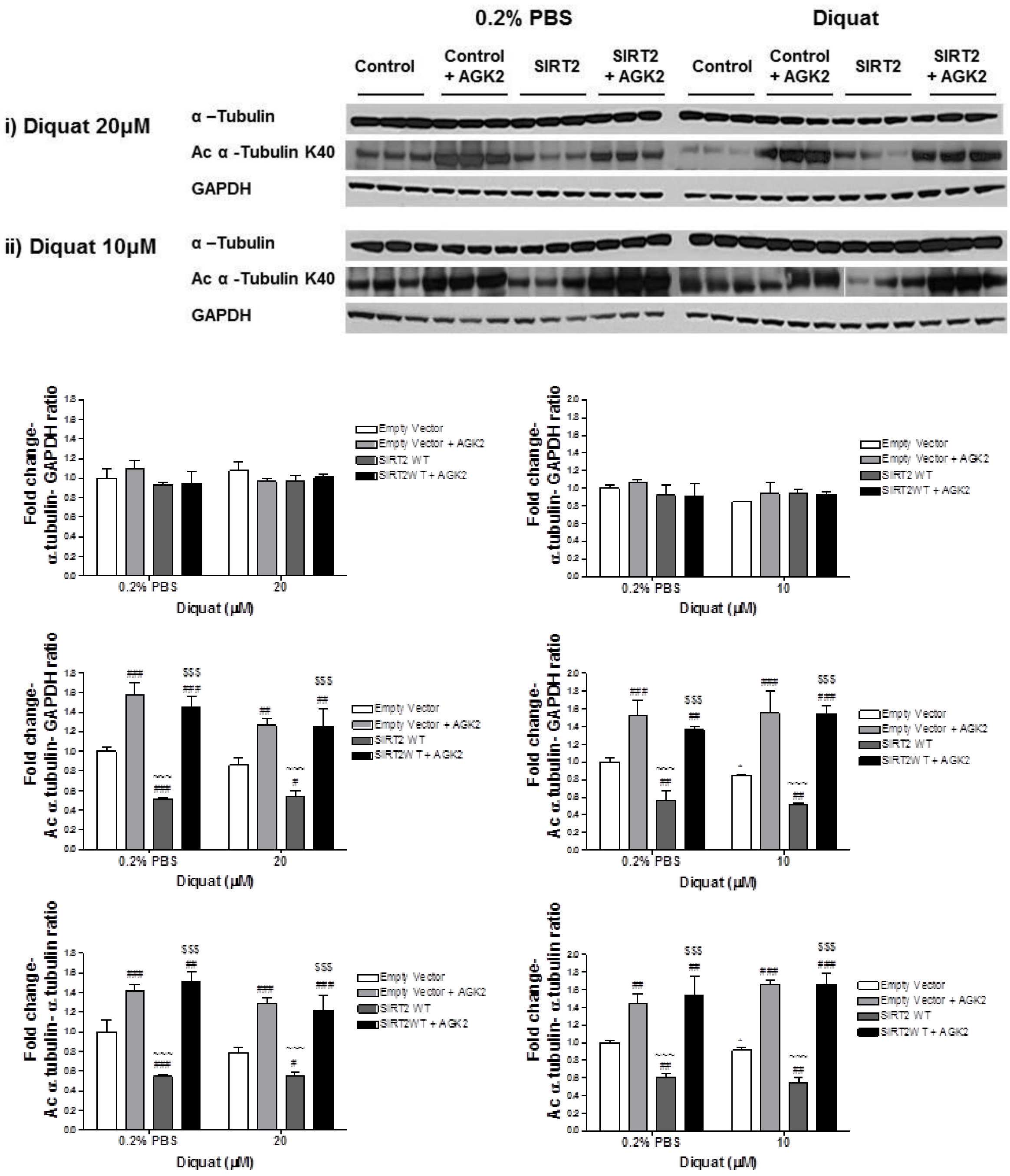

0.2% PBS
Diquat
Control
Control + AGK2
SIRT2
SIRT2 + AGK2
i) Diquat 20µM
ii) Diquat 10µM
α –Tubulin
Ac α -Tubulin K40
GAPDH
Fold change- α tubulin- GAPDH ratio
Fold change- Ac α tubulin- GAPDH ratio
Fold change- Ac α tubulin- α tubulin ratio
Empty Vector
Empty Vector + AGK2
SIRT2 WT
SIRT2WT + AGK2
0.2% PBS
20
10
Diquat (µM)

Rotenone

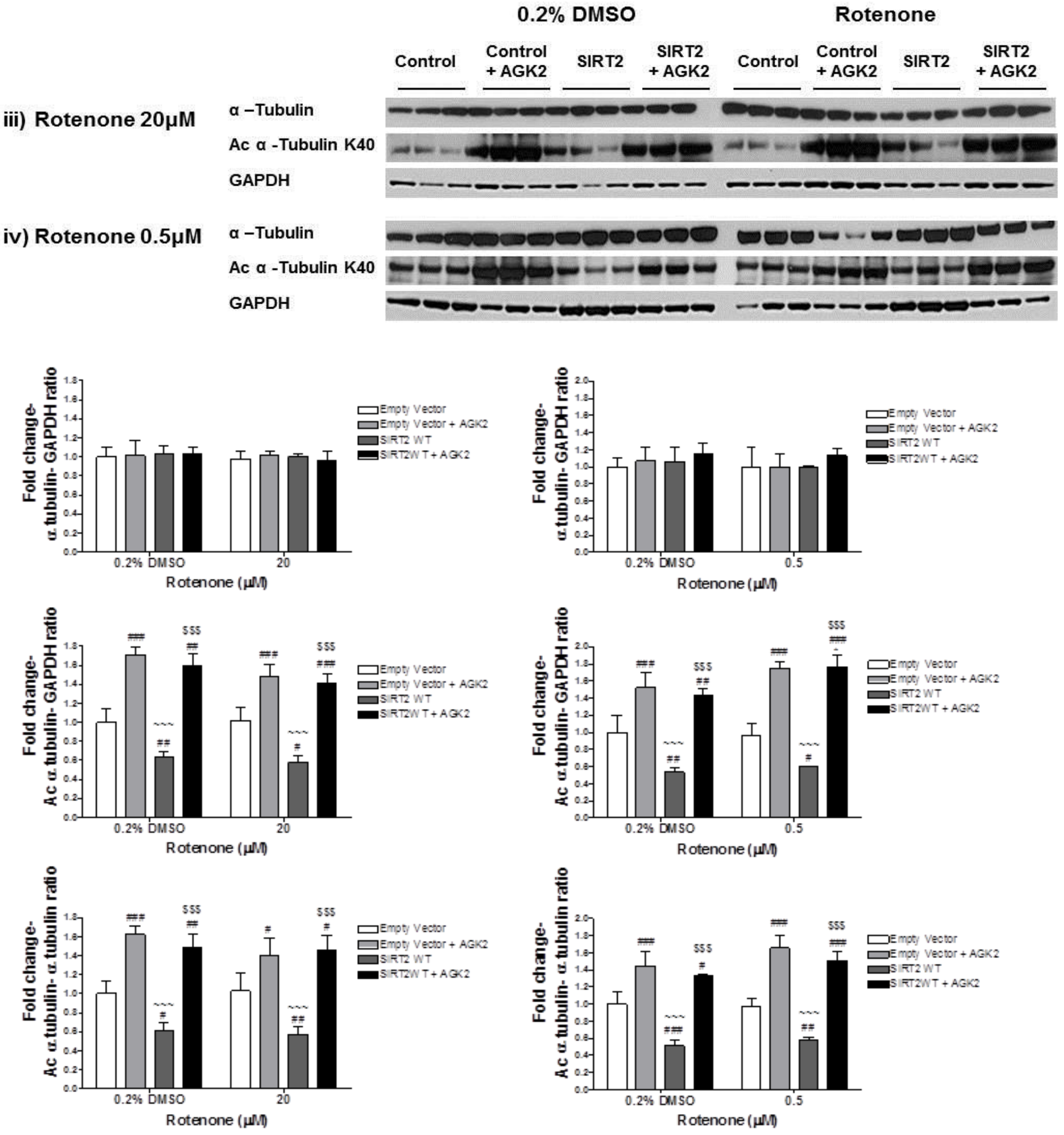

Supplementary Figure 2 AGK2 treatment and its effect on expression of α-tubulin in toxin treated SH-SY5Y cells.

SIRT2 was over-expressed in SH-SY5Y cells and control cells were transfected with empty vector following which one set of cells was treated with toxin alone and another with SIRT2 inhibitor AGK2 and toxin. Cells were harvested and the samples were probed for α-tubulin expression; a and c show the representative blot of α-tubulin, acetylated α-tubulin and GAPDH in cells treated with a) 20µM diquat or 10µM diquat and c) 20µM rotenone or 0.5µM rotenone. The graphs represent the data are presented as fold- untreated (+SD) in cells treated with b) 20µM diquat or 10µM diquat, d) 20µM rotenone or 0.5µM rotenone. Data presented are from three independent assays (n=3). *p<0.05 when compared to 0.2% PBS/DMSO, one-way ANOVA (Bonferroni corrected), ###p<0.001and ##p<0.01 when compared to empty vector treatment, ~~~p<0.001 and ~p<0.05 when compared to empty vector + AGK2 treatment and $$$p<0.001 and $$p<0.01 when compared to SIRT2 overexpressing cells, two-way ANOVA (Bonferroni-corrected).

3. Cellular location of SIRT2 under oxidative stress

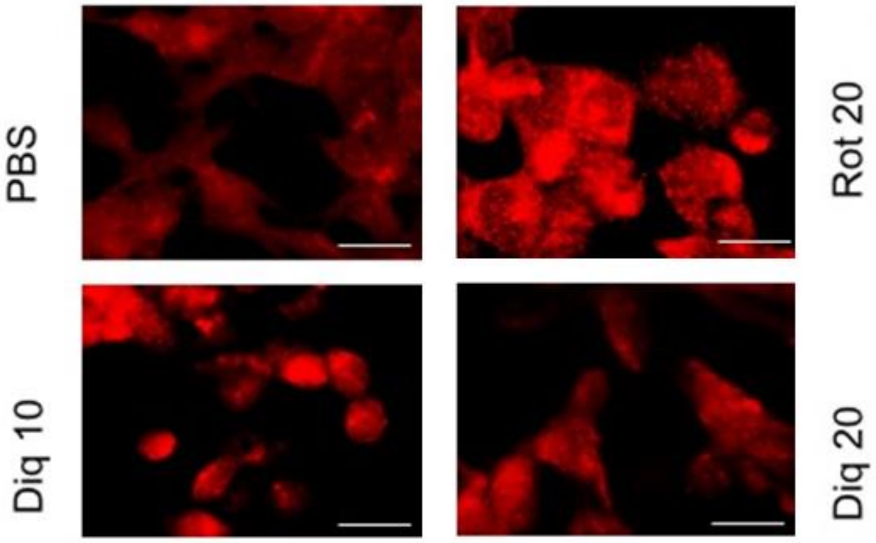

Supplementary Figure 3: SIRT2 localisation following oxidative stress.

Cells were treated with vehicle or with 10µM or 20µM diquat, or 20µM rotenone and stained to show localisation of SIRT2 within cells. Under basal conditions, SIRT2 showed a predominantly cytoplasmic localisation but following oxidative stress showed additional translocation to the nucleus.

4.Details of antibodies used in Western blotting and immunocytochemistry

	**Dilution**	**Incubation**	**Supplier**	**Secondary antibody**
**Primary Antibodies**				
SIRT2	1:5,000	ON at 4°C	Cell Signaling	Rabbit IgG-HRP
GAPDH- HRP	1:4,000	1 hour at RT	SantaCruz Biotechnology	-
Acetylated α-tubulin (K40)	1:100,000	ON at 4°C	Abcam	Mouse IgG- HRP
α-tubulin	1:100,000	ON at 4°C	Abcam	Rabbit IgG-HRP
SOD2	1:10,000	ON at 4°C	Abcam	Mouse IgG- HRP
**Secondary Antibodies**				
Rabbit VeriBlot	1:2, 000	30 minutes at RT	Abcam	-
Mouse VeriBlot	1:2, 000	30 minutes at RT	Abcam	-

Table 1 Details of antibodies used in Western blotting of SIRTs transfected and toxin treated SH-SY5Y cells. ON- Over night; RT- Room temperature

	**Dilution**	**Incubation**	**Supplier**	**Secondary Antibodies**
**Primary Antibodies**				
SIRT2	1:500	ON at 4°C	SantaCruz Biotechnology	Goat anti-rabbit Alexa Fluor® 594
Phospho-α-synuclein	1:500	ON at 4°C	Wako	Goat anti-mouse Alexa Fluor® 488
**Secondary Antibodies**				
Goat anti-rabbit Alexa Fluor® 594	1:500	1 hour at RT in dark	Thermo Fisher Scientific	-
Goat anti-mouse Alexa Fluor® 488	1:500	1 hour at RT in dark	Thermo Fisher Scientific	-

Table 2 Details of antibodies used in fluorescence immunocytochemistry of SIRTs transfected SH-SY5Y cells. ON- Over night; RT- Room temperature

5. SIRT Activity Assay

Samples and buffer preparation

Five millilitres of SIRT assay buffer was prepared with 500mM Tris-HCl, pH 8.0, containing 1.37 M sodium chloride, 27mM KCl, and 10mM $MgCl_2$. Diluted 1.5ml of stock in 13.5ml ddH_2O. Final concentration 50mM Tris-HCl, pH 8.0, containing 137mM sodium chloride, 2.7mM KCl, and 1mM $MgCl_2$ and was used in the assay and for diluting the reagents. Fifty millimolar solution of NAD^+ and 50mM of nicotinamide (NAM) were prepared in ddH2O. Fluorescent SIRT substrate p53 (379-382) Ac-RHKK(Ac)-AMC was synthesised by Cambridge Research Biolabs, UK. The stock was prepared as a 5mM solution in diluted SIRT Assay buffer i.e., 5.4mg in 1.37ml of SIRT assay buffer and was stored at -70°C. HDAC and SIRT inhibitors were diluted in diluted assay buffer- TSA (HDAC inhibitor) 10µM (0.1mM stock diluted in assay buffer) and AGK2 (SIRT2 inhibitor) 200µM (2mM stock diluted in assay buffer).

The efficiency of AGK2 as a potent SIRT2 inhibitor was analysed by treating the recombinant SIRT2 with different doses of AGK2 ranging from 1µM -60µM. As shown in Figure 3, treatment of recombinant SIRT2 with AGK2 showed diminished activity of SIRT2 with AGK2 1µM- 13%, 5µM- 35%, 10µM- 58%, 20µM- 74%, 40µM- 78% and 60µM- 79% of inhibition of SIRT2 activity was observed (Figure 4).

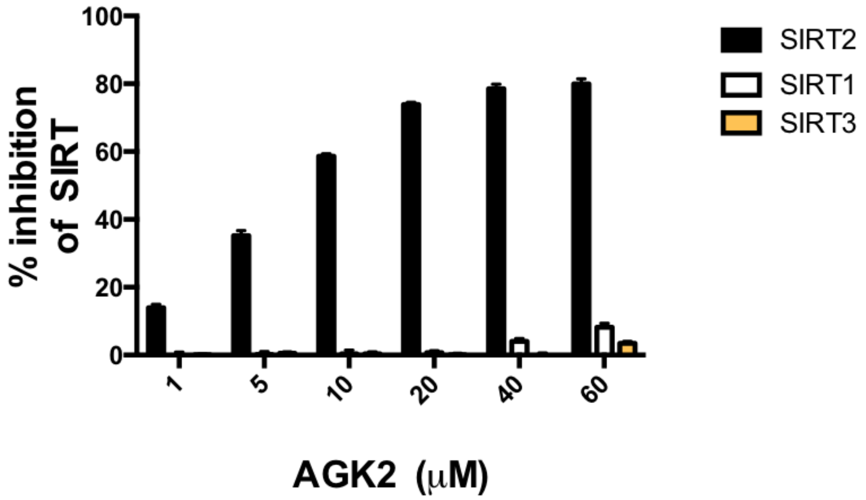

Supplementary Figure 4: Activity of AGK2 against Recombinant Sirtuins.

Recombinant SIRT1, SIRT2 or SIRT3 was used in the activity assay at 100ng/well and at a dose range of 1µM- 60µM for AGK2 in order to define the inhibitory characteristics of AGK2 as a specific SIRT2 inhibitor. AGK2 showed highly selective inhibition of SIRT2 up to 60µM.

The efficiency of the substrate Ac-RHKK(Ac)-AMC to measure SIRT activity was also analysed using recombinant SIRT1, SIRT2 and SIRT3. As shown in Figure 4, the substrate showed comparable deacetylase activity with the three recombinant SIRTs and no significant statistical difference was observed in activity of SIRT1, SIRT2 or SIRT3 (Supplementary Figure 5).

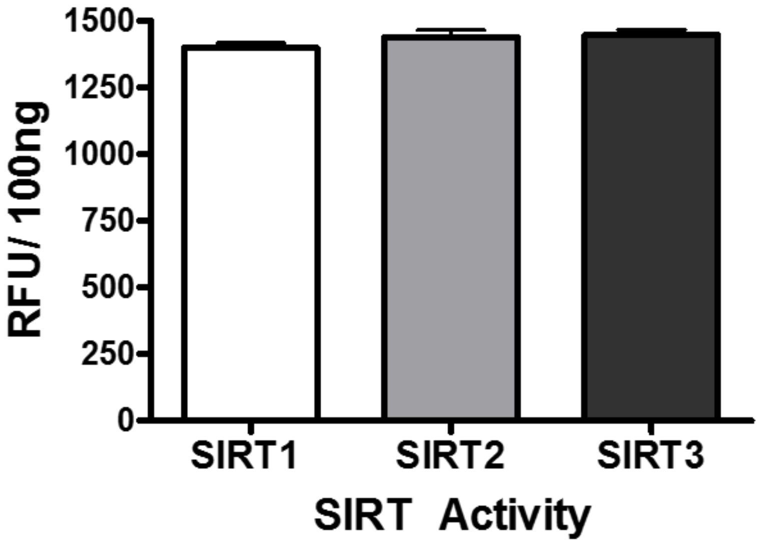

Supplementary Figure 5: SIRT activity assay with recombinant SIRTs using Fluorescent SIRT substrate p53 (379-382), Ac-RHKK(Ac)-AMC.

Recombinant SIRTs were used for the activity assay at 100ng/well to measure the deacetylase activity of SIRT1-SIRT3 towards the substrate. Recombinant sirtuins showed similar specific activity using the Ac-RHKK(Ac)-AMC substrate.

6. Cellular distribution of SIRT2 in the human brain

The location of SIRT2 was determined in different regions of the hippocampus– CA1, CA2, CA3 and CA4. In controls, SIRT2 was localised in the nucleus in CA1 neurones (Figure 6a) and CA2 neurones (Figure 6b) and was localised in both the nucleus and cytoplasm in CA3 neurones (Figure 6c) and CA4 neurones (Figure 6d), whereas in PD SIRT2 was present in the cytoplasm and occasionally present in the nucleus in CA1-4 (Figure 6 a-d). As in PD, in PDD cases, SIRT2 was localised both in the nucleus and cytoplasm in CA1 and CA2 but was prominently in the cytoplasm in CA3 and CA4 (Figure 6 a-d). In the DLB and AD, SIRT2 was present in the cytoplasm in CA1 (Figure 6a) neurones and in other CA subfields occasional nuclear localisation was observed but it was pre-dominantly present in the cytoplasm (Figure 6 b-d). Determination of SIRT2 sub-cellular localisation of SIRT2 in the cerebellum did not show any significant difference in disease groups and controls. In general, nuclear staining of Purkinje cells and granular neurones was observed in all groups (Figure 6e). SIRT2 did not show a staining pattern that could be associated to Lewy bodies. These results suggest that there was no significant effect of disease conditions on the location of SIRT2 within the cell.

a)

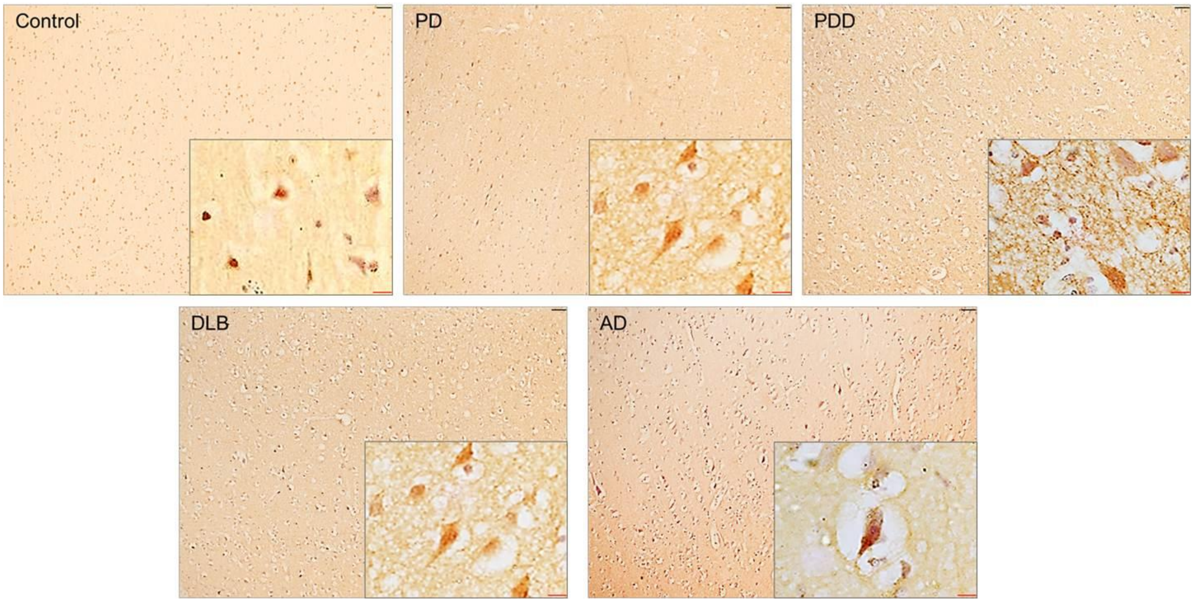

b)

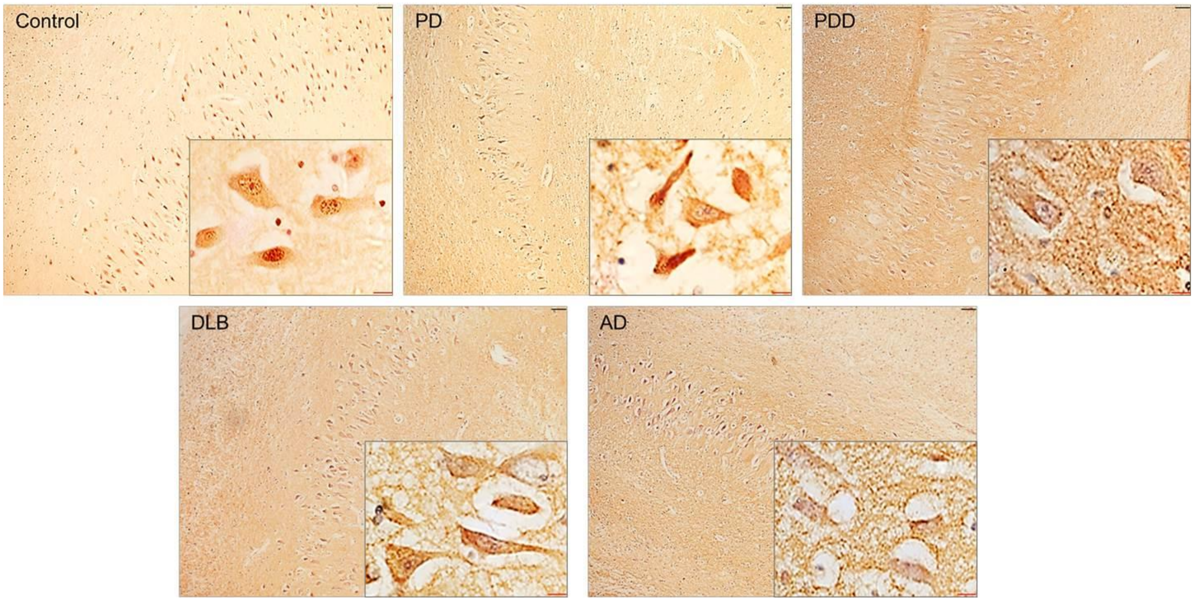

c)

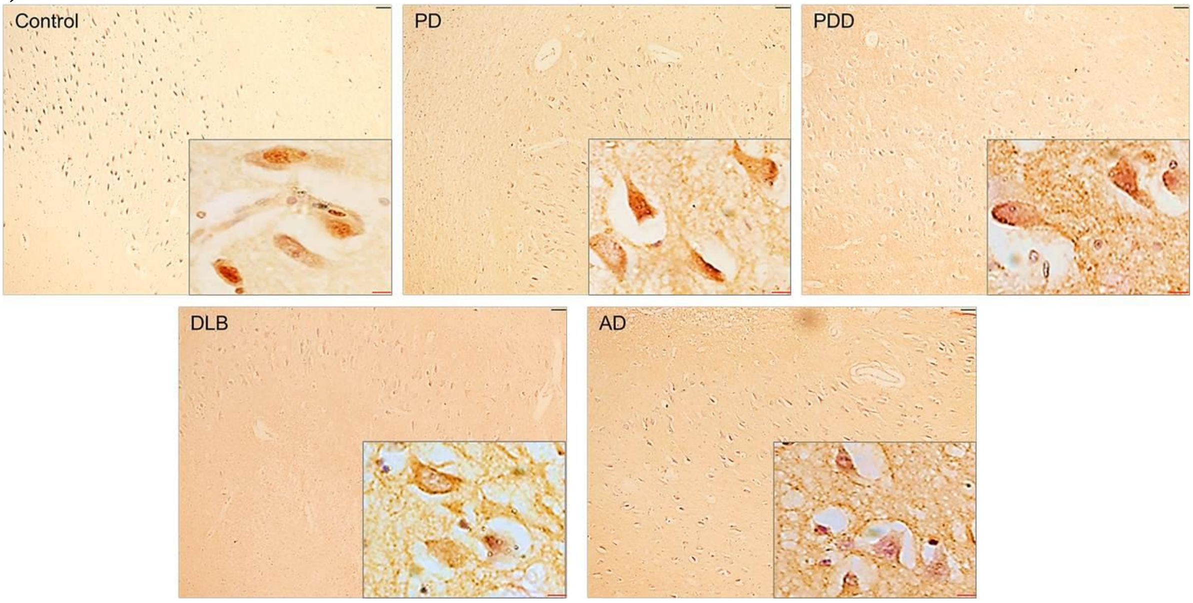

d)

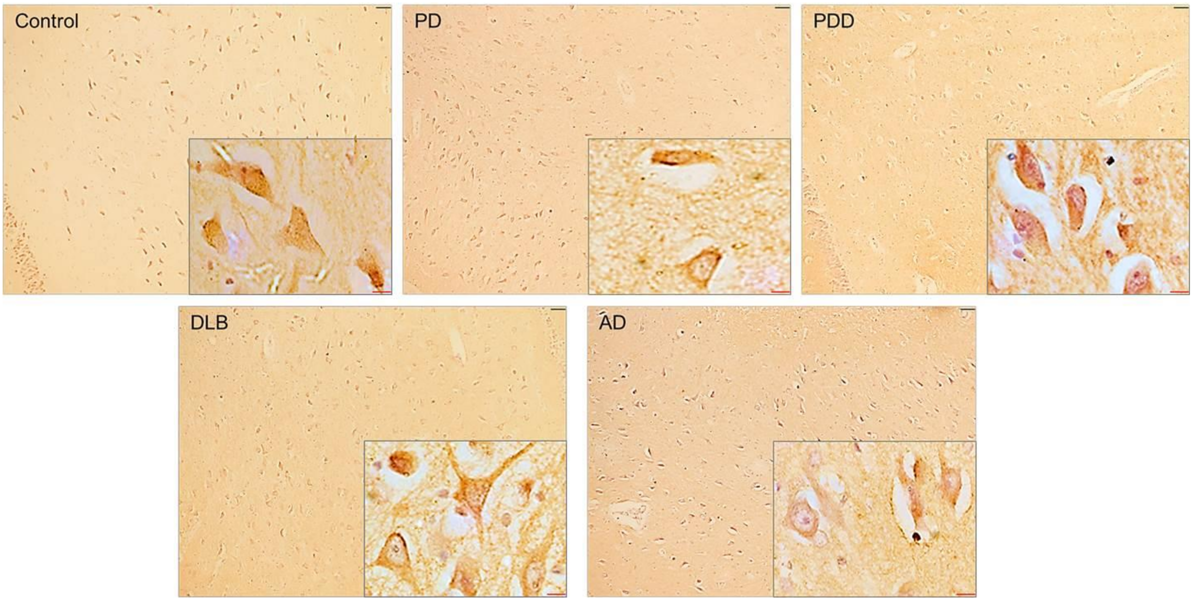

e)

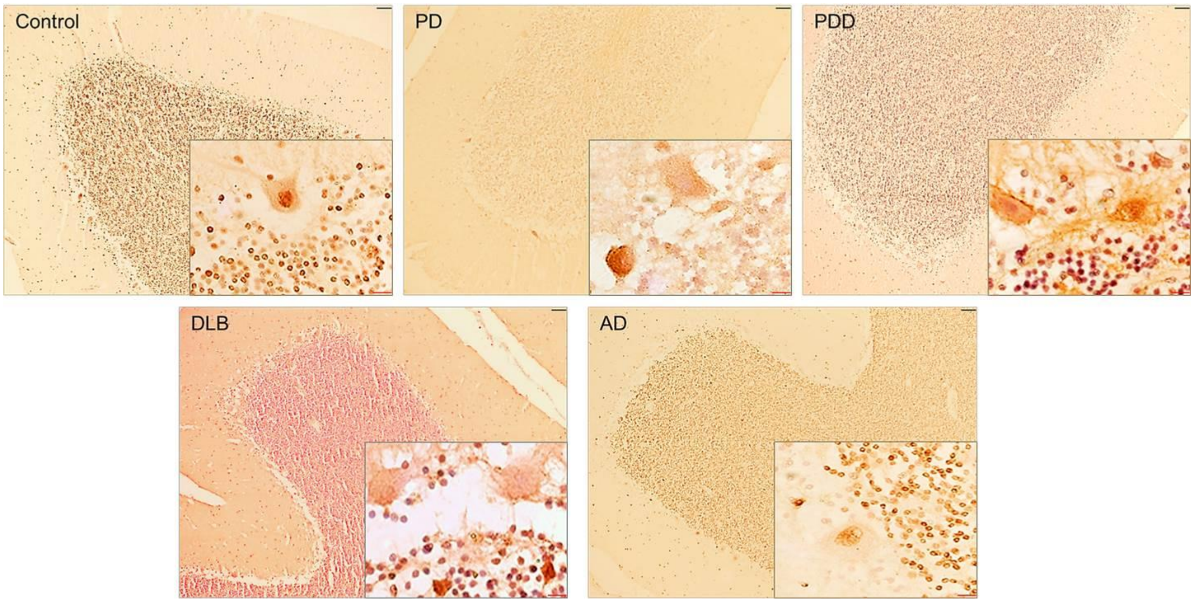

Supplementary Figure 6: Cellular distribution of SIRT2 in the Hippocampus and Cerebellum of Disease and Control Groups.

The images show the cellular localisation of SIRT2 in the grey matter of a) CA1, b) CA2, c) CA3, d) CA4 and e) cerebellum in PD, PDD, DLB, AD and control cases. The picture in inset is 63X oil immersion image overlaid on 10X image. Scale bars- black scale bar= 50µM and red scale bar= 20µM

**Temporal cortex (Superior Temporal Gyrus)**					
	**Control**	**PD**	**PDD**	**DLB**	**AD**
**General impression**	Nuclear and occasional cytoplasmic	Nuclear and occasional cytoplasmic	Nuclear and cytoplasmic	Nuclear and cytoplasmic	Cytoplasmic
**Hippocampus**					
	**Control**	**PD**	**PDD**	**DLB**	**AD**
**CA1**	Nuclear	More cytoplasmic and occasional nuclear	More cytoplasmic and occasional nuclear	Cytoplasmic	Cytoplasmic
**CA2**	Nuclear	More cytoplasmic and occasional nuclear	More cytoplasmic and occasional nuclear	Cytoplasmic	More cytoplasmic and bit nuclear
**CA3**	Nuclear and cytoplasmic	More cytoplasmic and occasional nuclear	Cytoplasmic	Cytoplasmic	More cytoplasmic and occasional nuclear
**CA4**	Nuclear and cytoplasmic	More cytoplasmic and occasional nuclear	Cytoplasmic	More cytoplasmic and occasional nuclear	More cytoplasmic and occasional nuclear
**Cerebellum**					
	**Control**	**PD**	**PDD**	**DLB**	**AD**
**General impression**	Nuclear in PCs, not so prominent glial cells or ASCs	Nuclear and cytoplasmic in PCs and granular layer	Cytoplasmic and nuclear both	Cytoplasmic PCs, nuclear granular	Nuclear in both

Table 3 Summary table presenting localisation of SIRT2 in different brain regions of PD, PDD, DLB, AD and control groups.
